# High Quality Growth of Cobalt Doped GaN Nanowires with Enhanced Ferromagnetic and Optical Response

**DOI:** 10.3390/ma13163537

**Published:** 2020-08-11

**Authors:** Mudassar Maraj, Ghulam Nabi, Khurram Usman, Engui Wang, Wenwang Wei, Yukun Wang, Wenhong Sun

**Affiliations:** 1 Research Center for Optoelectronic Materials and Devices, School of Physical Science and Technology, Guangxi University, Nanning 530004, China; mudassar@mail.ustc.edu.cn (M.M.); 1915301044@st.gxu.edu.cn (E.W.); 1814404039@st.gxu.edu.cn (W.W.); 20180116@gxu.edu.cn (Y.W.); 2 Department of Physics, University of Gujrat, Gujrat 50700, Pakistan; 3 International Academy of Optoelectronics, South China Normal University, Zhaoqing 526000, China; khurram.usman@zq-scnu.org; 4 Guangxi Key Laboratory of Processing for Non-Ferrous Metal and Featured Materials, Guangxi University, Nanning 530004, China

**Keywords:** GaN, semiconductor, cobalt doping, photoluminescence, ferromagnetism, spintronics

## Abstract

Group III–V semiconductors with direct band gaps have become crucial for optoelectronic and microelectronic applications. Exploring these materials for spintronic applications is an important direction for many research groups. In this study, pure and cobalt doped GaN nanowires were grown on the Si substrate by the chemical vapor deposition (CVD) method. Sophisticated characterization techniques such as X-ray diffraction (XRD), Scanning Electron Microscope (SEM), Energy Dispersive X-Ray Spectroscopy (EDS), Transmission Electron Microscopy (TEM), High-Resolution Transmission Electron Microscopy (HRTEM) and photoluminescence (PL) were used to characterize the structure, morphology, composition and optical properties of the nanowires. The doped nanowires have diameters ranging from 60–200 nm and lengths were found to be in microns. By optimizing the synthesis process, pure, smooth, single crystalline and highly dense nanowires have been grown on the Si substrate which possess better magnetic and optical properties. No any secondary phases were observed even with 8% cobalt doping. The magnetic properties of cobalt doped GaN showed a ferromagnetic response at room temperature. The value of saturation magnetization is found to be increased with increasing doping concentration and magnetic saturation was found to be 792.4 µemu for 8% cobalt doping. It was also depicted that the Co atoms are substituted at Ga sites in the GaN lattice. Furthermore N vacancies are also observed in the Co-doped GaN nanowires which was confirmed by the PL graph exhibiting nitrogen vacancy defects and strain related peaks at 455 nm (blue emission). PL and magnetic properties show their potential applications in spintronics.

## 1. Introduction

The spintronics industry aims to develop devices that can manipulate the spin of the electron as an additional degree of freedom, thereby providing a novel kind of electronic device which is replacing the traditional electronic sensors, Light-emitting diode (LED) displays, memory devices etc. This industry is progressing by exploring different research directions, such as the incorporation of magnetic ions in semiconductors [1], 2D materials for spintronic applications [2], Molecular Spin Gyroid structures [3], Giant Magneto-Resistance (GMR) based spintronic devices [4] etc.

Since the prediction of room temperature ferromagnetism by Deitl, the semiconductors, doped with ferromagnetic ions, are considered to be one of the sources of ferromagnetism [1]. These doped semiconductors are called dilute-magnetic semiconductors (DMSs) and have become important constituent of the spintronics industry. This new field of doped semiconductors offers a platform to work with the spin of polarized free charge carriers as an additional degree of freedom. The doping of magnetic ions into semiconductors, especially wide band gap semiconductors, has created the possibilities of integration of electrical, magnetic as well as optical properties of these versatile materials [5]. The main goal is to find a proper material that combines the desirable properties of ferromagnets and semiconductors. Among these, the GaN and ZnO-based diluted semiconductors (DMSs) with wide band gap characteristic properties, have gained considerable interest in theoretical predictions of ferromagnetism in these materials above room temperature. The magnetic response of GaN and ZnO-based DMSs was found to be strongly dependent on preparation techniques and the reported results varied noticeably from group to group [6]. Although ZnO is an auspicious candidate for spintronics devices, but high quality growth has limited its applications in DMSs. Additionally, the doping of p-type materials in ZnO is difficult and the fabrication of ZnO devices still has flaws [7]. On the other hand, nitride based semiconductors doped with transition metals (TMs) or rare earth elements for the sake of obtaining room temperature ferromagnetism have made tremendous progress [8].

Gallium nitride with a direct band gap (3.4 eV) is one of the ideal materials for ultraviolet (UV) and blue emitters, high-speed FETs and high-power electronic devices, which makes it a suitable candidate to develop spintronic devices. There are different reports on the doping of GaN with other TM and rare-earth elements, such as Mn [9,10,11], Cr [12,13,14], Gd [15,16], Dy [14], Eu [17], Co [18,19,20,21,22,23,24], V [25] and Fe [26,27,28,29,30,31]. These studies were mainly focused on bulk semiconductors, but the demand of next generation electronic devices with a minimum size but the same extraordinary properties as bulk material is increasing in the society. Among these, one dimension (1D) semiconductor nanostructures are more attractive for potential applications in the diminishment of electronic devices [32]. These 1D structures such as nanowires and nanotubes are the focus of research in DMSs for the miniaturization of spintronic devices [33,34,35,36]. For the practical applications of these nanostructure materials in spintronics devices, they must exhibit ferromagnetism with a critical temperature above room temperature. In this regard, the synthesis routes that grow high quality 1D structures, especially nanowires, are crucial to improve the morphology and physical properties.

1D structures of GaN have also been studied in recent years such as nanowires [37,38,39,40] and nanotubes [41,42]. Different dopants have been incorporated to optimize the physical proprieties of GaN based DMSs such as Mg [43,44,45], Mn [46], Fe [47], Cu [48], Si [49,50], Cr [51] and Tb [52], etc. However, the studies about Co-doped GaN nanowires are very few and even the existing results need to be optimized for a high Curie temperature, better structure, morphology, etc. [32,53,54].

Inspired by its growing intention in the scientific community, the study of the structural and magnetic properties of Co doped GaN with optimized morphology and better magnetic properties of cobalt doped GaN nanowires has been reported here. The enhanced magnetic properties ensure its applications for spintronics and the synthesis method employed here provides the route to obtain better morphology of these nanowires.

## 2. Experimental Procedure

**Figure 1:**
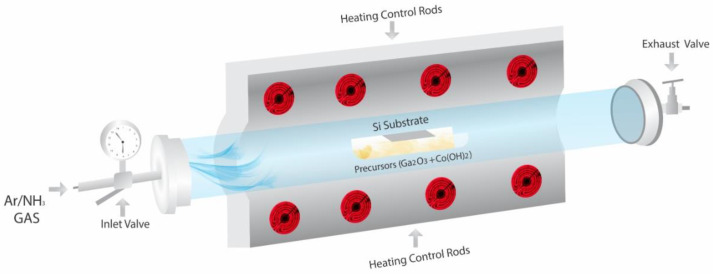
Schematic diagram of chemical vapor deposition (CVD) process.

Pure and Co-doped GaN nanowires were synthesized by a facile chemical vapor deposition (CVD) technique in the horizontal tube furnace (HTF) as shown in the schematic diagram of Figure 1. The mixture of Ga_2_O_3_ (99.9% pure) and Co(OH)_2_ (99.9% pure) powders with different ratios was put into the semicircle alumina boat. Ultrasonically cleaned and Hydrofluoric (HF) acid (5%) etched uncoated Si (100) substrate (size of 1 × 2 cm^2^) was positioned on top of the alumina tube already loaded with precursors materials in such a way that its shining side was downward. The distance between precursors and the Si substrate was about 0.5 cm. Both the pretreating and preheating techniques were employed simultaneously before the synthesis of pure and Co-doped GaN nanowires. In the pretreating technique, the precursors were pretreated five times with aqueous NH_3_ in a heating oven at 100 °C for 10 min each time. After each heating, a few drops of aqueous NH_3_ were added and agitated with a glass rod for complete mixing. Subsequently, a preheating technique was used as mentioned elsewhere [47].

The alumina semicircle tube loaded with pretreated/preheated precursors and Si substrates was shifted into the horizontal tube furnace (HTF). After that, the HTF was sealed from both ends and the environmental residual contents were evacuated by a mechanical evacuating pump. Moreover, intensive flushing with high purity (99.99% pure) NH_3_ gas was also performed. Subsequently, the flow rate of NH_3_ gas was adjusted to 50 sccm and the furnace was gradually heated at a ramp rate of 10 °C/min to obtain the required reaction temperature of 1200 °C, which was maintained for 120 min. After the completion of the reaction, the furnace was allowed to cool down naturally to room temperature in the presence of NH_3_ gas flow to avoid oxidation. The light yellow products gathered on the substrate were analyzed for further characterizations and physical properties studies.

The structure of the synthesized samples was characterized by the X-ray diffraction technique (Philips X’Pert Pro MPD, Malvern Panalytical’s Ltd, Malvern, UK) with a Cu-Kα radiation source (λ = 0.15418 nm), while the morphology was studied by field emission scanning electron microscopy (FESEM, S-4800, Hitachi, Tarrytown, NY, USA) equipped with an energy dispersive X-ray spectrometer (EDX), transmission electronic microscope and high-resolution transmission electronic microscope (FES TECNAI F20, FEI, Hillsborough, OR, USA). The optical properties (photoluminescence) at room temperature were studied using a PL spectrum from a LabRAM HR Evolution (HORIBA, Paris, France). The ferromagnetic response was investigated using magnetic hysteresis (MH) loops obtained through a vibrating sample magnetometer (Lake Shore 7400, Lake Shore Cryotronics Inc., Westville, OH, USA).

## 3. Structural Characterization

**Figure 2:**
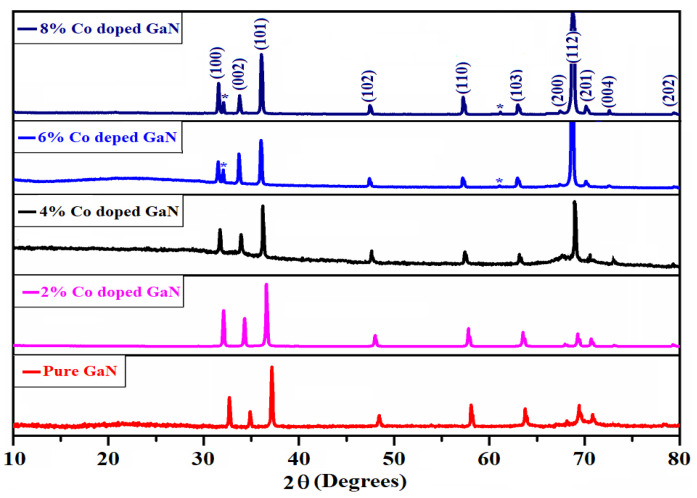
X-ray diffraction of pure and cobalt doped (2%, 4%, 6% and 8%) GaN nanowires.

The crystal structure and phases of pure and Co-doped GaN nanowires (GNNWs) were analyzed using an X-ray diffraction (XRD) technique and the results are depicted in Figure 2. The crystal structures of the synthesized samples are presented for the 2θ range 10–80°. To identify the crystalline quality as a result of cobalt doping, the doped samples were also compared with pure GaN XRD peak positions and intensity variation.

These XRD patterns of pure GaN and the gradual increase of 2%, 4%, 6% and 8% Co doped GNNWs were matched with JCPDS Card No.076-0703. Figure 2 shows sharp peaks with higher intensity to confirm the better crystallinity of the pure and doped GaN samples. It was observed that the crystal structure of GaN was hexagonal in nature and their lattice parameter values (a = 3.162 Å, c = 5.189 Å) are in good agreement with the literature [55]. The XRD spectra showed pure and single phases of the nanowires and no impurity peak was found in the analysis. Miller indices of the hexagonal GNNWs are also marked in the graphs relevant to the peaks. It is noted that the doping of cobalt in GaN resulted in the shifting of peaks to the smaller angles which caused an increase in lattice parameters, as reported in the literature [24]. Furthermore, the doping with cobalt did not modify the diffraction profile of GaN. The good dispersion of the cobalt ions in the crystalline lattice of the GaN or the small fraction of dopant addition prevented the observation of signals referable to the cobalt incorporation in the crystalline phases. This experimental evidence suggests that, independent of the synthesis method, cobalt doping does not modify typical wurtzite structure of GaN [56,57].

## 4. SEM and EDS

**Figure 3:**
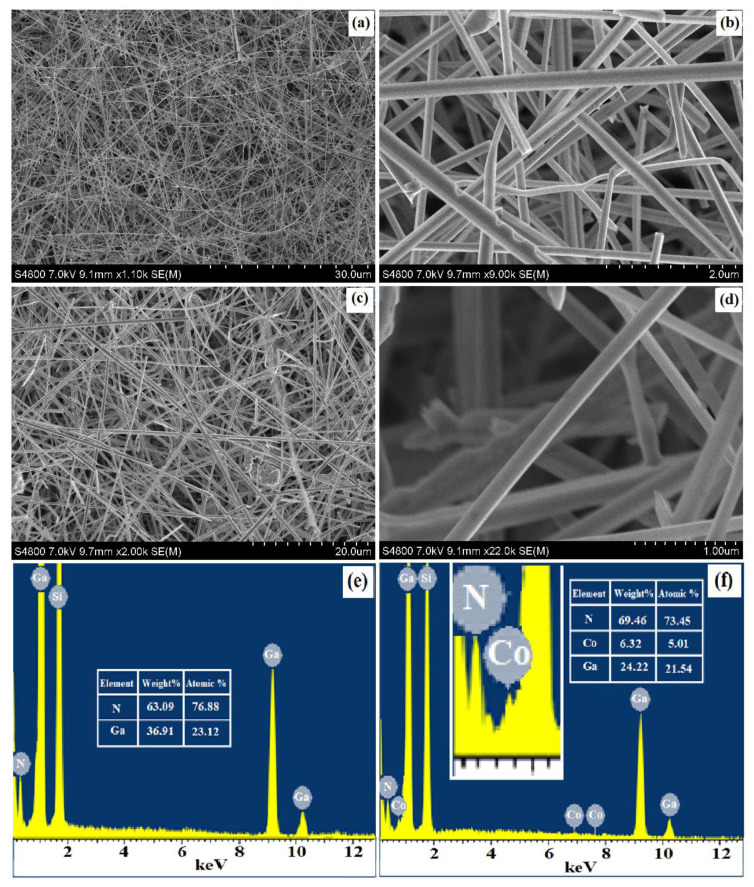
SEM micrographs of (**a**) pure GaN nanowires, (**b**) their magnified image, (**c**) 6% cobalt doped GaN nanowires and (**d**) 6% cobalt doped GaN nanowires magnified image. Also (**e**) and (**f**) are the EDS analyses of pure and cobalt doped GaN nanowires with their element ratios, respectively. The inset in Figure 3f shows the closer view of cobalt incorporation.

Morphological and elemental analyses of pure and Co-doped GaN samples were carried out using Field Emission scanning Electron Microscope (FESEM), equipped with EDS, as depicted in Figure 3. Highly concentrated pure GaN nanowires are shown in Figure 3a while Figure 3b shows its magnified image. These GaN nanowires were found to be of a diameter in the range of 60–200 nm, whereas the lengths were in the microns.

Their corresponding EDS analysis is shown in Figure 3e confirming the distribution of gallium nitride on the Si substrate. The percentage ratio of the Ga and N in the GaN are depicted in the inset of Figure 3e. The cobalt doped GaN nanowires are shown in Figure 3c, and the magnified image of 6% cobalt doped nanowires is shown in Figure 3d. The size of the doped nanowires was also found to be almost same as that of pure GaN nanowires. Figure 3d confirms that the Co-doped GaN nanowires were also smooth and there was no tube-like or belt-like structures in the doped samples. The confirmation of cobalt doping in GaN on the Si substrate is represented by the EDS analysis, as shown in the Figure 3f. The percentage ratio of the Ga, N and Co are depicted in the inset of Figure 3f. These SEM images illustrate that a large number of Co:GNNWs was developed which covered the Si substrate. The percentages of Si in Figure 3e,f are excluded for a better understanding. The smaller peaks of cobalt (Co) at 0.776 keV are related to Lα (Lα = 0.776 keV) and their height is consistent with the literature and EDX analysis [54]. The surface of the nanowires was found to be smooth, homogenous and free of domain boundaries. The SEM micrographs exhibit that most of the Co:GNNWs had a relatively smooth surface and indicated the high yield of NWs.

**Figure 4:**
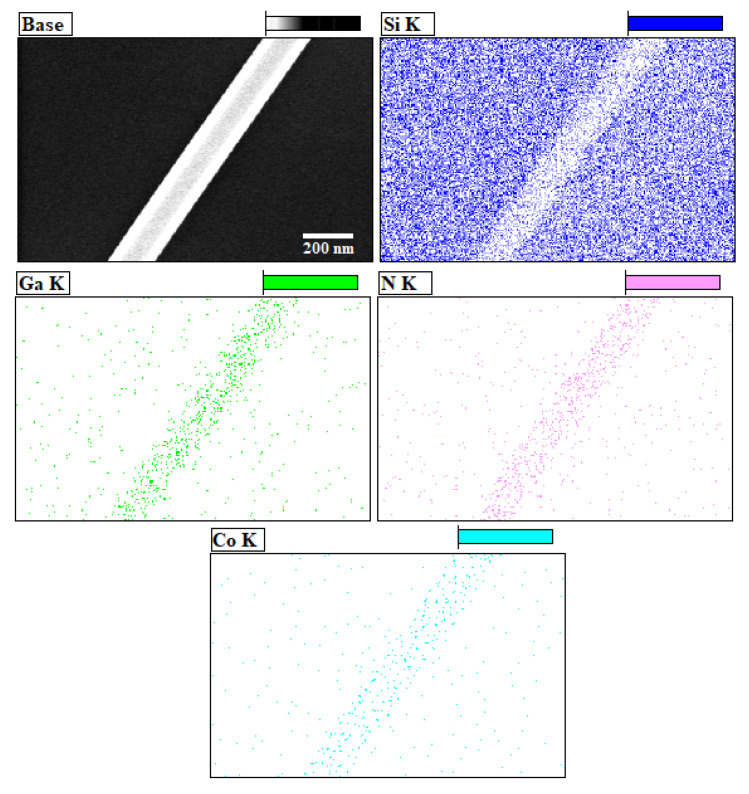
EDS mapping for dispersion of different elements involved in the synthesis of nanowires.

The elemental analysis of the doped nanowires was also verified by the EDS mapping which is presented in Figure 4. A highly magnified portion of a single nanowire was chosen for this mapping, as shown in the base diagram of Figure 4 along with the mapping of individual constituents—Si, Ga, N and cobalt. This figure shows the uniform dispersion of the elements during the synthesis process and confirms the good quality of the grown samples.

## 5. TEM Analysis

**Figure 5:**
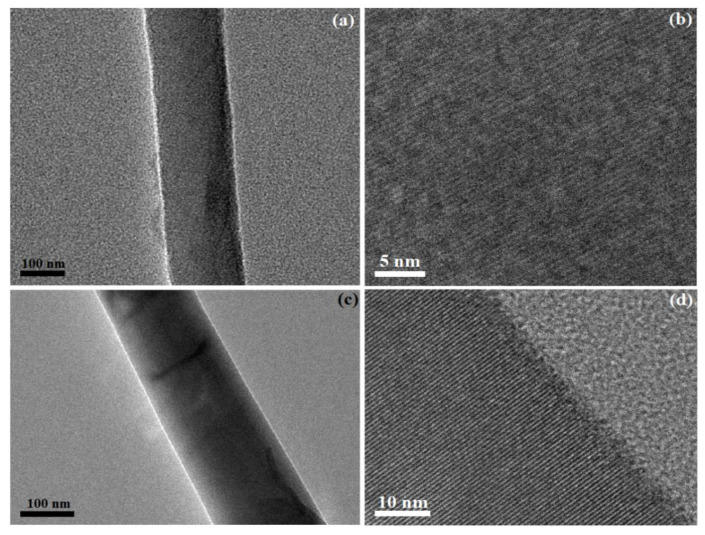
(**a**) TEM of pure GaN nanowire, (**b**) HRTEM of pure GaN nanowire, (**c**) TEM of the 6% Co-doped GaN nanowire, (**d**) HRTEM 6% Co-doped GaN nanowire.

Figure 5 shows typical TEM and HRTEM images of pure and cobalt dope GNNWs. The TEM images of pure GaN nanowire is shown in Figure 5a which reveals that NWs were single crystalline with uniform thicknesses and smooth surfaces along their entire length. Figure 5b shows HRTEM of pure GaN nanowires and observations showed that the interplanar distance was 5.17 Å and this value was consistent with the XRD results.

The TEM image of the 6% doped cobalt is shown in Figure 5c. These doped nanowires were found to be free of any secondary phases along their entire lengths. These observations were consistent with previous findings for Co-doped GaN NWs, while Figure 5d represents HRTEM of doped nanowires. The HRTEM lattice image shows negligible defects in the lattice planes. The interval between the two fringes was 5.20 Å which was little bit larger than the plane distance of the pure GaN nanowires. The HRTEM images in parallel to SEM images further confirmed the smooth and controlled the synthesis of the Co-doped GaN nanowires. The high resolution TEM images, shown in Figure 5b,d, indicate that the lattice fringes were well-separated without forming any pairing structure.

## 6. Magnetic Properties

**Figure 6:**
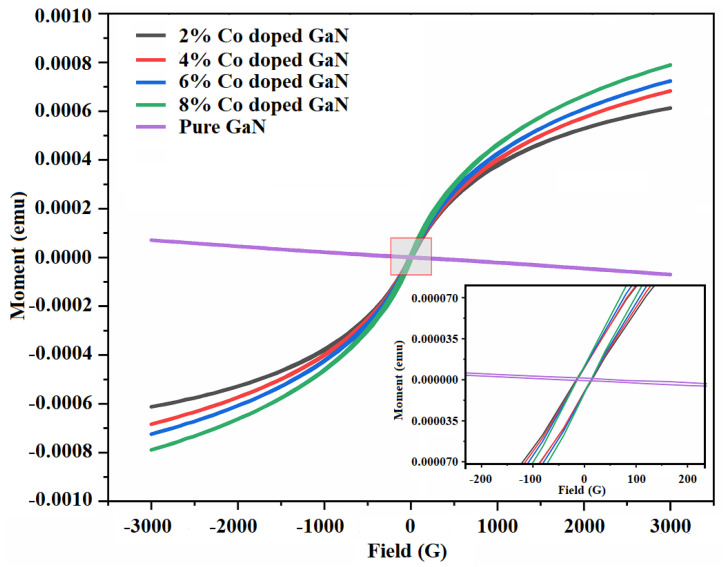
M versus H curves for pure and Co:GNNWs (2%, 4%, 6% and 8%).

The magnetic properties of Co-doped GaN nanowires with 2%, 4%, 6% and 8% were determined by vibrating sample magnetometer (VSM) at room temperature (RT). Figure 6 shows their magnetic hysteresis loops, indicating that the Co-doped GaN nanowires were ferromagnetic at room temperature. As a comparison, the hysteresis loop of pure GaN nanowires is also depicted which showed diamagnetic behavior at RT. In all magnetic data presented here, the contributions from the silicon substrate were removed. The ferromagnetism at 300 K was clearly shown by the remanence and coercivity suggesting that the Curie temperature (Tc) was at least 300 K. It is clear that the saturation magnetization increased with the increase in the concentration of cobalt doping. Their corresponding saturation magnetization, retentivity and coercivity are shown in Table 1.

The observed magnetic behavior may be attributed to the exchange interaction between the localized magnetic dipole moments of the magnetic ions and the free delocalized charge of current carriers [32].

The comprehensive comparison of magnetic properties of different dopants incorporation in GaN nanowires is presented in Table 2. We saw that saturation magnetization was almost three times greater than those reported in [32] and the calculated values were sufficient to be exploited for spintronics applications [53,54]. The reported work was mainly focused on the enhanced morphology of the synthesized nanowires, which was far better than the already reported cobalt doped nanowires.

## 7. Photoluminescence (Optical Properties)

Ultraviolet (UV) light used to excite the product is obtained from xenon lamp and its excitation wavelength has been set at 325 nm. The room-temperature photoluminescence (PL) emission spectrum of

**Figure 7:**
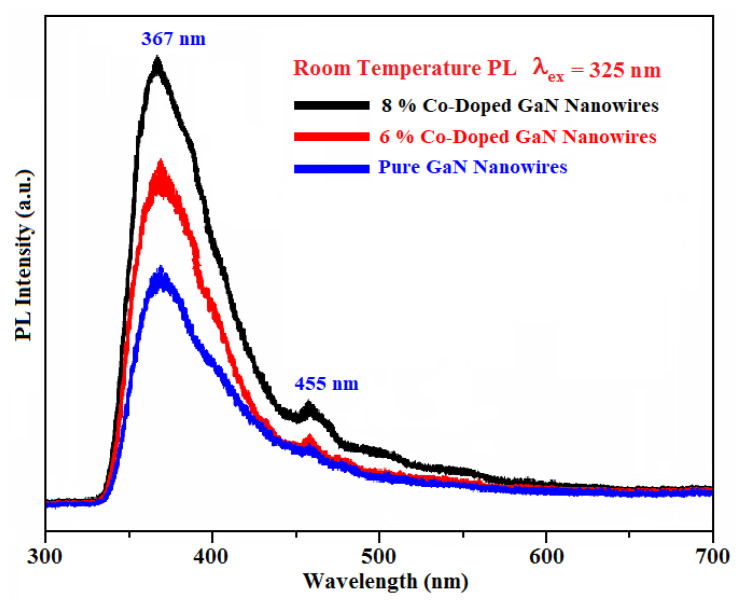
Photoluminescence of pure and cobalt doped (6% and 8%) GaN nanowires.

The PL technique provided better information about the localized defects and impurity incorporation effects within the nanostructures. Therefore, to study the optical response of these nanowires, PL analysis of pure GaNNWs and Co-doped GaNNWs were studied at room temperature and the resulting spectrum is shown in Figure 7. The pure GaN, 6% and 8% Co-doped GaN NWs were excited with 325 nm He-Cd laser with a power of 0.0016 mW. Pure GaN NWs exhibited a single emission peak at 367 nm corresponding to a band edge transition of GaN agreeing with the literature [60]. The 6% and 8% Co-doped GaN NW excited with same laser caused band-edge (BE) emission at 368.85 nm (3.361 eV) for 6% and 370.26 nm (3.348) for 8% cobalt doping, causing a small red shift due to the inclusion of cobalt in GaN. This type of minor red shifting in band edge peak was mostly attributed to the strains occurring because of Co and other impurity (defects) in the GaN structures. Besides this, some weaker peaks were also present in the spectra, specifically at 455 nm (blue emission) for 6% and 8% cobalt doping. These extra peaks in GaN provided the confirmation of Co dopant-related energy levels in the Co-doped GaN samples.

Generally, the peak at 455 nm was ascribed to the nitrogen vacancies in GaN that may also arise due to strain and defects in the GaN structures [61]. Such kind of blue emission was also observed in the Fe doped GaN nanowires and is consistent with the literature [47]. Another important observation in the PL studies was that, with increasing the contents of cobalt in GNNWs, the defect related peak at 455nm was more prominent as compared to the less doped Cobalt in GaN. This also explains that, with the addition of Co, more defects could be created, and more blue emission could be attained. The photoluminescence response of different dopants in GaN nanowires is presented in Table 3. In this study, the Co-doped GaN nanowires showed nitrogen vacancy defects and strains at 455 nm (blue emission), which may be used for blue LEDs.

## 8. Conclusions

We have successfully synthesized Co-doped GaN nanowires by a chemical vapor deposition technique, using pretreated Ga_2_O_3_ and Co(OH)_2_ as precursors. The structural and morphological analyses show that pure and Co-doped GaN nanowires are single crystalline and have a size in the range of 60–200 nm diameter and without any secondary phases (Ga_2_O_3_ or Co_2_O_3_), as confirmed by XRD. The small shift in the XRD peaks proved the incorporation of Co into the GaN lattice which were in good agreement with the PL results. The EDS analysis displayed only Ga, N and Co peaks in the respective samples with no notable impurities. The room temperature magnetic properties exhibited that Co doped GNMSs are ferromagnetic in nature and an increasing trend in magnetism was observed with increasing Co contents. In PL, along with the near band edge emission at 367 nm, a relatively weaker peak in the blue emission region is ascribed to the nitrogen vacancies. A smaller shift in the peak position is attributed to the presence of Co, giving rise to the strain shifting of peaks. It is believed that Co-doped GNNWs may serve as a promising candidate for optoelectronics (blue LEDs) and spintronic devices. We assertively conclude that these nanowires are good candidates for the fabrication of next generation smart opto-electronic devices.

## Figures and Tables

**Table 1:** The effect of dopant concentration on magnetization of Co-doped GaN nanowires.

Concentration	Mr (µemu)	Ms (µemu)	Hc (G)
8%	12.2	792.4	17.2
6%	11.6	771.2	12.1
4%	10.1	684.5	10.4
2%	12.9	614.8	10.9

**Table 2:** Comparison of magnetic properties of different doped magnetic ions in GaN nanowires.

Dopant	Concentration	Mr (emu)	Ms (emu)	Hc	Ref.
Fe	20%	0.49 × 10^−6^	13.54 × 10^−6^	42.76 G	[47]
Pd	1.63%	-	8.5 × 10^−6^	148 Oe	[58]
Mn	5%	12.9 × 10^−6^	1.48 × 10^−6^	400 Oe	[59]
Co	1.25%	31.62 × 10^−6^	273.57 × 10^−6^	108.16 G	[32]
Co	1.55%	2.84	6.67 × 10^−3^	269.35 Oe	[53]
Co	8%	12.2 × 10^−6^	792.4 × 10^−6^	10.9 G	This work

**Table 3:** Comparison of PL properties various dopants in GaN nanowires.

Dopant	B.E Emission	Defects	Ref.
Fe	36 9nm	467 nm	[47]
Mg	358 nm	426 nm	[44]
Mn	401.4 nm	700 nm	[46]
Co	360.1 nm	No defect	[53]
Co	363.85 nm	421.57 nm	[54]
Co	367 nm	455 nm	This work
